# A second ortho­rhom­bic polymorph of 3,5-diphenyl-4*H*-1,2,4-triazol-4-amine

**DOI:** 10.1107/S1600536809033807

**Published:** 2009-08-29

**Authors:** Ya-Wen Zhang, Jian-Quan Wang, Lin Cheng

**Affiliations:** a Department of Chemistry and Chemical Engineering, State Key Laboratory of Coordination Chemistry, Nanjing University, Nanjing 211189, People’s Republic of China; b Department of Chemistry and Chemical Engineering, Southeast University, Nanjing 211189, People’s Republic of China

## Abstract

The present crystal structure is the second ortho­rhom­bic polymorph of the title compound, C_14_H_12_N_4_. Whereas the structure in *Pnma* with *Z*′ = 0.5 is already known [Ikemi *et al.* (2002 ▶). *Heterocycl. Commun.* **8**, 439–442], the present structure crystallizes in the space group *Pbca* with *Z*′ = 1. The dihedral angle between the two phenyl rings is 23.5 (4)° and the dihedral angles between central ring and the phenyl rings are 41.0 (3) and 26.3 (5)°. In the 4-amino-1,2,4-trizole fragment, the C=N distances are 1.321 (3) and 1.315 (3) Å, which are much shorter than the C—N distances of 1.367 (3) and 1.357 (3) Å. In the crystal, adjacent mol­ecules are linked by N—H⋯N hydrogen bonds.

## Related literature

For 4-amino-1,2,4-triazoles derivatives, see: Beckmann & Brooker (2003 ▶); Collin *et al.* (2003 ▶). For the other polymorph, see: Ikemi *et al.* (2002 ▶).
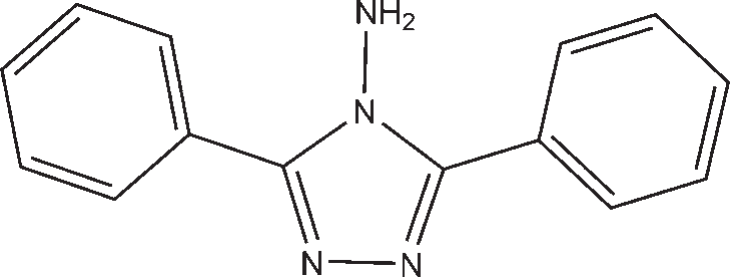


## Experimental

### 

#### Crystal data

C_14_H_12_N_4_
*M* *_r_* = 236.28
Orthorhombic, 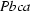

*a* = 7.5521 (9) Å
*b* = 11.2309 (14) Å
*c* = 28.278 (3) Å
*V* = 2398.4 (5) Å^3^
*Z* = 8
Mo *K*α radiation
μ = 0.08 mm^−1^
*T* = 293 K
0.30 × 0.28 × 0.25 mm

#### Data collection

Bruker SMART CCD diffractometer
Absorption correction: multi-scan (*SADABS*; Sheldrick, 2000 ▶) *T*_min_ = 0.976, *T*_max_ = 0.980
9495 measured reflections
2307 independent reflections
1613 reflections with *I* > 2σ(*I*)
*R*_int_ = 0.029

#### Refinement

*R*[*F* ^2^ > 2σ(*F* ^2^)] = 0.056
*wR*(*F* ^2^) = 0.161
*S* = 1.07
2307 reflections
164 parameters
H-atom parameters constrained
Δρ_max_ = 0.16 e Å^−3^
Δρ_min_ = −0.18 e Å^−3^

### 

Data collection: *SMART* (Bruker, 2000 ▶); cell refinement: *SAINT* (Bruker, 2000 ▶); data reduction: *SAINT*; program(s) used to solve structure: *SHELXTL* (Sheldrick, 2008 ▶); program(s) used to refine structure: *SHELXTL*; molecular graphics: *SHELXTL*; software used to prepare material for publication: *SHELXTL*.

## Supplementary Material

Crystal structure: contains datablocks I, global. DOI: 10.1107/S1600536809033807/bt5044sup1.cif

Structure factors: contains datablocks I. DOI: 10.1107/S1600536809033807/bt5044Isup2.hkl

Additional supplementary materials:  crystallographic information; 3D view; checkCIF report

## Figures and Tables

**Table 1:** Hydrogen-bond geometry (Å, °)

*D*—H⋯*A*	*D*—H	H⋯*A*	*D*⋯*A*	*D*—H⋯*A*
N4—H4*B*⋯N2^i^	0.95	2.17	3.117 (2)	177

Symmetry code: (i) 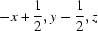.

**Fig. 1.**
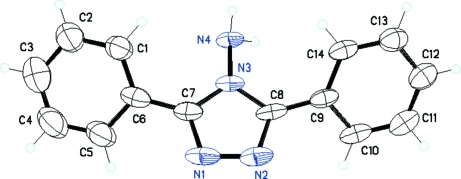
Structure of the title compound with 30% thermal ellipsoids.

**Fig. 2.**
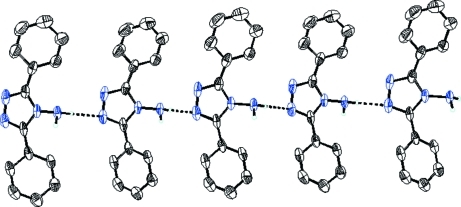
The one-dimensional hydrogen bonding network of the title compound.

## supplementary crystallographic information

### Comment

The derivatives of 4-amino-1,2,4-triazoles have been extensively investigated in in medicinal chemistry and agricultural chemistry (Collin *et al.*, 2003). They are a type of multidentate ligands in coordination chemistry (Beckmann *et al.* 2003). Herein, we report the crystal structure of 3,5-diphenyl-4*H*-1,2,4-triazol-4-amine.

The title compound, C_14_H_12_N_4_, is a 4-amino-3,5-disubstituted-1,2,4-trizole compound and with a dihedral angle between the two phenyl rings of  23.5 (4) °. In the 4-amino-1,2,4-trizole fragment, the C=N distance is 1.321 (3) and 1.315 (3) Å, which are much shorter than the C—N distances of 1.367 (3) and 1.357 (3) Å. In the crystal, adjacent molecules are linked by N—H···N hydrogen bonds into a one-dimensional chain with N···N distance 3.117 (3) Å. The crystal structure of the title compound  is a second orthorhombic polymorph. Whereas the structure in Pnma with Z'=0.5 is already known(Ikemi *et al.* 2002), the present structure has the space group Pbca with Z'=1.

### Experimental

A mixture solution of the benzonitrile (0.103 g, 1.0 mmol), 50% NH_2_NH_2_.H_2_O (3 ml) and ethanol (2 ml) was heated in a 15 ml Teflon-lined autoclave at 100 ° for 3 days, followed by slow cooling (5 ° h-1) to room temperature. The colorless block crystals were collected by filtration washed with water, then dried and collected in 11.9% yield (0.014 g) based on benzonitrile.

### Refinement

H atoms bonded to N atoms were located in a difference map with the restraint of N—H = 0.95 Å. Other H atoms were positioned geometrically and refined using a riding model with C—H = 0.93 Å and with *U*_iso_(H) = 1.2 *U*_iso_(C).

### Crystal data

**Table:**

C_14_H_12_N_4_	*F*(000) = 992
*M**_r_* = 236.28	*D*_x_ = 1.309 Mg m^−^^3^
Orthorhombic, *P**b**c**a*	Mo *K*α radiation, λ = 0.71073 Å
Hall symbol: -P 2ac 2ab	Cell parameters from 1617 reflections
*a* = 7.5521 (9) Å	θ = 2.9–27.7°
*b* = 11.2309 (14) Å	µ = 0.08 mm^−^^1^
*c* = 28.278 (3) Å	*T* = 293 K
*V* = 2398.4 (5) Å^3^	Block, colorless
*Z* = 8	0.30 × 0.28 × 0.25 mm

### Data collection

**Table:**

Bruker SMART CCD diffractometer	2307 independent reflections
Radiation source: fine-focus sealed tube	1613 reflections with *I* > 2σ(*I*)
graphite	*R*_int_ = 0.029
φ and ω scans	θ_max_ = 26.0°, θ_min_ = 2.9°
Absorption correction: multi-scan (*SADABS*; Sheldrick, 2000)	*h* = −8→8
*T*_min_ = 0.976, *T*_max_ = 0.980	*k* = −8→13
9495 measured reflections	*l* = −34→34

### Refinement

**Table:**

Refinement on *F*^2^	Secondary atom site location: difference Fourier map
Least-squares matrix: full	Hydrogen site location: inferred from neighbouring sites
*R*[*F*^2^ > 2σ(*F*^2^)] = 0.056	H-atom parameters constrained
*wR*(*F*^2^) = 0.161	*w* = 1/[σ^2^(*F*_o_^2^) + (0.075*P*)^2^ + 0.3879*P*] where *P* = (*F*_o_^2^ + 2*F*_c_^2^)/3
*S* = 1.07	(Δ/σ)_max_ < 0.001
2307 reflections	Δρ_max_ = 0.16 e Å^−^^3^
164 parameters	Δρ_min_ = −0.18 e Å^−^^3^
0 restraints	Extinction correction: *SHELXTL* (Sheldrick, 2008), Fc^*^=kFc[1+0.001xFc^2^λ^3^/sin(2θ)]^-1/4^
Primary atom site location: structure-invariant direct methods	Extinction coefficient: 0.0018 (7)

### Special details

**Table:**

Geometry. All e.s.d.'s (except the e.s.d. in the dihedral angle between two l.s. planes) are estimated using the full covariance matrix. The cell e.s.d.'s are taken into account individually in the estimation of e.s.d.'s in distances, angles and torsion angles; correlations between e.s.d.'s in cell parameters are only used when they are defined by crystal symmetry. An approximate (isotropic) treatment of cell e.s.d.'s is used for estimating e.s.d.'s involving l.s. planes.
Refinement. Refinement of *F*^2^ against ALL reflections. The weighted *R*-factor *wR* and goodness of fit *S* are based on *F*^2^, conventional *R*-factors *R* are based on *F*, with *F* set to zero for negative *F*^2^. The threshold expression of *F*^2^ > σ(*F*^2^) is used only for calculating *R*-factors(gt) *etc*. and is not relevant to the choice of reflections for refinement. *R*-factors based on *F*^2^ are statistically about twice as large as those based on *F*, and *R*- factors based on ALL data will be even larger.

### Fractional atomic coordinates and isotropic or equivalent isotropic displacement parameters (Å^2^)

**Table:**

	*x*	*y*	*z*	*U*_iso_*/*U*_eq_	
C1	0.1186 (4)	0.1019 (2)	0.34391 (9)	0.0903 (8)	
H1A	0.1495	0.0470	0.3671	0.108*	
C2	0.0906 (5)	0.0634 (3)	0.29855 (10)	0.1183 (11)	
H2A	0.1035	−0.0168	0.2912	0.142*	
C3	0.0438 (5)	0.1425 (5)	0.26392 (13)	0.1355 (13)	
H3A	0.0215	0.1166	0.2333	0.163*	
C4	0.0307 (6)	0.2600 (4)	0.27534 (14)	0.1452 (16)	
H4A	0.0020	0.3147	0.2519	0.174*	
C5	0.0585 (5)	0.2991 (3)	0.32027 (12)	0.1164 (11)	
H5A	0.0482	0.3799	0.3270	0.140*	
C6	0.1018 (3)	0.2208 (2)	0.35588 (9)	0.0750 (7)	
C7	0.1304 (3)	0.26686 (18)	0.40343 (9)	0.0672 (6)	
C8	0.1462 (2)	0.28566 (15)	0.48031 (9)	0.0646 (6)	
C9	0.1367 (2)	0.26276 (16)	0.53087 (8)	0.0623 (6)	
C10	0.0670 (3)	0.35010 (19)	0.56046 (10)	0.0766 (7)	
H10A	0.0238	0.4206	0.5476	0.092*	
C11	0.0618 (3)	0.3326 (2)	0.60841 (11)	0.0893 (8)	
H11A	0.0159	0.3914	0.6280	0.107*	
C12	0.1239 (3)	0.2287 (2)	0.62747 (10)	0.0901 (8)	
H12A	0.1197	0.2171	0.6600	0.108*	
C13	0.1924 (3)	0.1416 (2)	0.59877 (10)	0.0839 (7)	
H13A	0.2339	0.0711	0.6120	0.101*	
C14	0.1998 (3)	0.15788 (18)	0.55063 (9)	0.0694 (6)	
H14A	0.2471	0.0988	0.5313	0.083*	
N1	0.1804 (3)	0.37701 (15)	0.41296 (9)	0.0858 (7)	
N2	0.1900 (3)	0.38864 (15)	0.46152 (9)	0.0816 (6)	
N3	0.1098 (2)	0.20701 (13)	0.44473 (6)	0.0597 (5)	
N4	0.0340 (2)	0.09288 (13)	0.44985 (6)	0.0639 (5)	
H4B	0.1214	0.0327	0.4533	0.077*	
H4C	−0.0467	0.0933	0.4756	0.077*	

### Atomic displacement parameters (Å^2^)

**Table:**

	*U*^11^	*U*^22^	*U*^33^	*U*^12^	*U*^13^	*U*^23^
C1	0.109 (2)	0.0712 (17)	0.0906 (18)	−0.0055 (14)	−0.0115 (14)	0.0118 (13)
C2	0.157 (3)	0.103 (2)	0.095 (2)	−0.014 (2)	−0.0136 (18)	0.0066 (18)
C3	0.147 (3)	0.166 (4)	0.094 (2)	0.000 (3)	−0.009 (2)	0.014 (2)
C4	0.180 (4)	0.142 (4)	0.114 (3)	0.052 (3)	0.017 (2)	0.053 (3)
C5	0.143 (3)	0.094 (2)	0.112 (2)	0.0370 (19)	0.033 (2)	0.0354 (19)
C6	0.0566 (13)	0.0589 (13)	0.1096 (18)	0.0088 (10)	0.0127 (11)	0.0270 (13)
C7	0.0547 (13)	0.0442 (12)	0.1026 (17)	0.0067 (9)	0.0109 (10)	0.0087 (11)
C8	0.0410 (11)	0.0299 (10)	0.1228 (18)	−0.0006 (7)	0.0057 (10)	−0.0089 (10)
C9	0.0407 (11)	0.0405 (10)	0.1057 (16)	−0.0069 (8)	0.0007 (9)	−0.0121 (10)
C10	0.0526 (13)	0.0444 (12)	0.133 (2)	−0.0024 (9)	0.0074 (12)	−0.0186 (12)
C11	0.0712 (16)	0.0771 (18)	0.120 (2)	−0.0073 (13)	0.0126 (14)	−0.0343 (16)
C12	0.0805 (18)	0.0828 (18)	0.1069 (19)	−0.0079 (14)	0.0000 (13)	−0.0208 (15)
C13	0.0754 (16)	0.0659 (15)	0.110 (2)	−0.0029 (12)	−0.0134 (13)	−0.0073 (13)
C14	0.0521 (13)	0.0455 (12)	0.1107 (19)	−0.0004 (9)	−0.0064 (11)	−0.0167 (11)
N1	0.0767 (14)	0.0379 (10)	0.143 (2)	−0.0021 (9)	0.0250 (12)	0.0139 (11)
N2	0.0750 (14)	0.0409 (10)	0.1290 (18)	−0.0081 (8)	0.0187 (11)	−0.0036 (10)
N3	0.0452 (9)	0.0324 (8)	0.1015 (13)	0.0009 (6)	0.0045 (8)	0.0037 (8)
N4	0.0606 (11)	0.0310 (8)	0.1000 (13)	−0.0052 (7)	0.0100 (8)	0.0013 (7)

### Geometric parameters (Å, °)

**Table:**

C1—C2	1.370 (4)	C8—C9	1.454 (3)
C1—C6	1.383 (3)	C9—C14	1.388 (3)
C1—H1A	0.9300	C9—C10	1.393 (3)
C2—C3	1.368 (5)	C10—C11	1.371 (4)
C2—H2A	0.9300	C10—H10A	0.9300
C3—C4	1.362 (5)	C11—C12	1.368 (4)
C3—H3A	0.9300	C11—H11A	0.9300
C4—C5	1.361 (5)	C12—C13	1.373 (3)
C4—H4A	0.9300	C12—H12A	0.9300
C5—C6	1.377 (3)	C13—C14	1.375 (3)
C5—H5A	0.9300	C13—H13A	0.9300
C6—C7	1.457 (3)	C14—H14A	0.9300
C7—N1	1.321 (3)	N1—N2	1.381 (3)
C7—N3	1.356 (3)	N3—N4	1.411 (2)
C8—N2	1.315 (2)	N4—H4B	0.9499
C8—N3	1.367 (3)	N4—H4C	0.9498
			
C2—C1—C6	121.3 (3)	C14—C9—C8	121.92 (18)
C2—C1—H1A	119.4	C10—C9—C8	119.0 (2)
C6—C1—H1A	119.4	C11—C10—C9	120.3 (2)
C3—C2—C1	120.4 (3)	C11—C10—H10A	119.9
C3—C2—H2A	119.8	C9—C10—H10A	119.9
C1—C2—H2A	119.8	C12—C11—C10	120.1 (2)
C4—C3—C2	118.6 (4)	C12—C11—H11A	119.9
C4—C3—H3A	120.7	C10—C11—H11A	119.9
C2—C3—H3A	120.7	C11—C12—C13	120.3 (3)
C5—C4—C3	121.5 (3)	C11—C12—H12A	119.9
C5—C4—H4A	119.2	C13—C12—H12A	119.9
C3—C4—H4A	119.2	C12—C13—C14	120.4 (2)
C4—C5—C6	120.9 (3)	C12—C13—H13A	119.8
C4—C5—H5A	119.6	C14—C13—H13A	119.8
C6—C5—H5A	119.6	C13—C14—C9	119.8 (2)
C5—C6—C1	117.4 (3)	C13—C14—H14A	120.1
C5—C6—C7	118.9 (3)	C9—C14—H14A	120.1
C1—C6—C7	123.7 (2)	C7—N1—N2	107.83 (19)
N1—C7—N3	108.7 (2)	C8—N2—N1	107.78 (18)
N1—C7—C6	124.3 (2)	C7—N3—C8	106.89 (17)
N3—C7—C6	126.98 (19)	C7—N3—N4	125.81 (18)
N2—C8—N3	108.8 (2)	C8—N3—N4	126.36 (17)
N2—C8—C9	124.41 (19)	N3—N4—H4B	112.0
N3—C8—C9	126.83 (17)	N3—N4—H4C	109.5
C14—C9—C10	119.1 (2)	H4B—N4—H4C	111.8

### Hydrogen-bond geometry (Å, °)

**Table:**

*D*—H···*A*	*D*—H	H···*A*	*D*···*A*	*D*—H···*A*
N4—H4B···N2^i^	0.95	2.17	3.117 (2)	177

Symmetry codes: (i) −*x*+1/2, *y*−1/2, *z*.
